# Trends in Human Papillomavirus Testing Among Patients With Oropharyngeal Cancer

**DOI:** 10.1001/jamanetworkopen.2025.23917

**Published:** 2025-07-29

**Authors:** Katie M. Carlson, Nana-Hawwa Abdul-Rahman, Rebecca A. Deek, Alexis Cenname, Wesley Cai, Kathryn Demanelis, Dan P. Zandberg, Kevin J. Contrera, Jose P. Zevallos, Jeremy Martinson, Angela L. Mazul

**Affiliations:** 1 Department of Otolaryngology–Head and Neck Surgery, University of Pittsburgh School of Medicine, Pittsburgh, Pennsylvania; 2 Cancer Epidemiology and Prevention Program, UPMC Hillman Cancer Center, Pittsburgh, Pennsylvania; 3 Department of Biostatistics and Health Data Science, University of Pittsburgh School of Public Health, Pittsburgh, Pennsylvania; 4 University of Pittsburgh Health Sciences Library System, Pittsburgh, Pennsylvania; 5 Division of Hematology and Oncology, Department of Medicine, University of Pittsburgh, Pittsburgh, Pennsylvania; 6 UPMC Hillman Cancer Center, University of Pittsburgh Medical Center, Pittsburgh, Pennsylvania; 7 Department of Medical Oncology, University of Pittsburgh Medical Center, Pittsburgh, Pennsylvania; 8 Department of Infectious Diseases and Microbiology, University of Pittsburgh School of Public Health, Pittsburgh, Pennsylvania

## Abstract

**Importance:**

Due to the prognostic value of human papillomavirus (HPV) status, HPV testing for oropharyngeal squamous cell carcinoma (OPSCC) has been recommended since 2012. However, recent HPV testing rates and associated sociodemographic and clinical factors are unknown.

**Objective:**

To examine HPV testing rates stratified by sociodemographic and clinical factors and factors associated with increased likelihood of not receiving HPV testing from 2013 to 2021.

**Design, Setting, and Participants:**

In this hospital-based cross-sectional study, the National Cancer Database (NCDB) was queried in 2024 for any patient diagnosed with OPSCC from 2013 to 2021, creating a retrospective cohort of 135 756 individuals. Two subsets were formed based on year of diagnosis: 2013-2017 and 2018-2021.

**Exposures:**

Sociodemographic characteristics (ie, age, race and ethnicity, sex, insurance status, zip code median income, and zip code educational attainment) and clinical factors (ie, year of diagnosis, subsite, overall stage, and facility type).

**Main Outcomes and Measures:**

The outcome variable was HPV testing status (tested vs not tested). Differences in HPV testing were assessed using χ^2^ and Mann-Whitney *U* testing. Trends in HPV testing were examined with annual percentage change longitudinally from 2013 to 2021. Factors associated with the increased probability of not receiving HPV testing were identified using Poisson regression.

**Results:**

The total population was 135 756 patients with OPSCC (7.3% Black, 3.9% Hispanic, 86.2% White, 2.6% other; 83.4% male). From 2013 to 2017, a total of 70 911 patients were diagnosed with OPSCC (82.9% male and 17.1% female). Of these, 50 494 (71.2%) were tested for HPV (median [IQR] age, 60 [47-73] years), whereas 20 417 (28.8%) were not tested for HPV or had unknown testing status (median [IQR], 62 [48-76] years). From 2018 to 2021, a total of 64 845 patients were diagnosed with OPSCC (83.9% male and 16.1% female). Of these, 60 184 (92.8%) were tested for HPV (median [IQR] age, 63 [50-76] years), whereas 4661 (7.2%) were not tested for HPV or had unknown testing status (median [IQR], 64 [49-79] years). In a multivariable analysis, patients with OPSCC who were diagnosed from 2018 to 2021 at community (prevalence ratio [PR], 1.36; 95% CI, 1.20-1.54), comprehensive community (PR, 1.20; 95% CI, 1.11-1.29), or integrated network (PR, 1.17; 95% CI, 1.06-1.28) cancer programs were more likely not to be tested for HPV compared with academic or research programs. Patients with OPSCC diagnosed from 2018 to 2021 of Black (PR, 1.18; 95% CI, 1.05-1.33) or other race (PR, 1.39; 95% CI, 1.17-1.66) were more likely not to be tested compared with White patients.

**Conclusions and Relevance:**

This cross-sectional study found substandard HPV testing rates and HPV testing disparities for patients with OPSCC in the NCDB from 2013 to 2021. These findings underscore the need for increased scrutiny of HPV testing practices, particularly at community cancer programs and among patients of races other than White, to ensure that OPSCC guidelines are met.

## Key Points

**Question:**

How have human papillomavirus (HPV) testing rates for oropharyngeal cancer changed from 2013 to 2021, and is testing associated with sociodemographic and clinical factors?

**Findings:**

In this cross-sectional study of 135 756 patients with oropharyngeal cancer, 28.8% of those diagnosed from 2013 to 2017 and 7.2% of those diagnosed from 2018 to 2021 were not tested for HPV or had unknown status. In adjusted analysis, Black race and community facility type were associated with lower likelihood of HPV testing compared with White race and academic or research facility type.

**Meaning:**

The results of this study suggest that further research is warranted to determine barriers to HPV testing and interventions to increase HPV testing.

## Teaser

This cross-sectional study of patients with oropharyngeal cancer examines human papillomavirus testing rates stratified by sociodemographic and clinical factors and factors associated with increased likelihood of not undergoing testing.

## Introduction

The epidemiology of oropharyngeal squamous cell carcinoma (OPSCC) has recently shifted, with an increase in human papillomavirus (HPV)–positive cases and a decrease in HPV-negative cases. In 2018, the Centers for Disease Control and Prevention declared that OPSCC had surpassed cervical cancer as the most common HPV-associated cancer in the US.^1^ This conclusion was based on data from 1999 to 2015, which showed that incidence rates for OPSCC in the US increased by 2.7% per year in men and 0.8% per year in women.^1^

Given that HPV-positive and HPV-negative OPSCC are distinct, clinical management of the disease has evolved. In 2010, a landmark study^2^ using data from a randomized clinical trial revealed that patients with HPV-positive OPSCC had a 3-year overall survival rate of 82.4% compared with 57.1% for HPV-negative patients. Furthermore, after adjusting for age, race and ethnicity, tumor and nodal stage, tobacco use, and treatment type, HPV-positive patients had a 58% reduction in the risk of death compared with HPV-negative patients.^2^ In 2012, the National Comprehensive Cancer Network (NCCN) recommended that all incident cases of OPSCC be tested for the presence of HPV^3^ because HPV status is considered an independent prognostic factor for OPSCC.^4^ Ongoing deescalation clinical trials are evaluating the use of lower doses of chemotherapy and radiotherapy for treating HPV-positive OPSCC to mitigate treatment-associated toxic effects.^5^ In 2018, the *American Joint Committee on Cancer Staging Manual*, eighth edition (*AJCC 8*), incorporated HPV status (determined through p16 immunohistochemical analysis) into the staging of OPSCC.^6^ Under this edition, many HPV-positive OPSCC cases previously classified as advanced (stages III or IV) were reclassified to earlier stages, reflecting the improved outcomes typically associated with HPV-positive cancers.^6^

Before 2018, two studies^7,8^ found that HPV testing rates in the years after the NCCN recommendation were substandard, especially among patients in underserved and minority populations. Since implementing *AJCC 8*, no studies have explored trends or determinants from 2018 onward. The current study examines HPV testing rates stratified by sociodemographic and clinical factors and factors associated with increased likelihood of not receiving HPV testing between 2013 and 2021. We hypothesized that patients of higher socioeconomic status would have higher rates of HPV testing.

## Methods

A Participant User File from the National Cancer Database (NCDB) was used to create a retrospective cohort of 135 756 individuals diagnosed with OPSCC from 2013 to 2021 (eFigure 1 in Supplement 1). The dataset was filtered only to include cases that correspond to OPSCC (*International Classification of Diseases for Oncology, Third Edition* [*ICD-O-3*] codes: C019 [base of tongue], C090, C091, C098, C099, and C024 [tonsil], and C100, C101, C102, C103, C104, C108, C109, C142 [other subsite]; and histology codes: 8070-8086). Two subsets (years of diagnosis 2013-2017 and 2018-2021) were constructed to address coding differences in the HPV testing status variable in the NCDB starting in 2018. Furthermore, using 2018 as the cutoff year allowed us to investigate HPV testing rates and associated factors starting in the year that *AJCC 8* was implemented. This study was reviewed and determined to be exempt from approval and informed consent by the institutional review board of the University of Pittsburgh because the NCDB patient data were deidentified and HIPAA-adherent. This study follows the Strengthening the Reporting of Observational Studies in Epidemiology (STROBE) reporting guideline for cross-sectional studies.

### Exposures

Exposure variables included demographic, socioeconomic, clinical, and hospital factors. Race and ethnicity as designated by the NCDB based on patient-reported data were categorized as Hispanic, non-Hispanic Black, non-Hispanic White, and other (self-identified, including otherwise not specified). Insurance status included uninsured, private insurance or managed care, Medicaid, Medicare, and other government insurance. For educational attainment, patients were categorized based on the percentage of adults 25 years and older in their zip code who did not possess a high school diploma (≥17.6%, 10.9%-17.5%, 6.3%-10.8%, and <6.3%). For median income, patients were categorized into levels based on the median annual household income in their zip code (<$40 227, $40 227-$50 353, $50 354-$63 332, and≥$63 333). Educational attainment and median income were determined from the 2016 five-year American Community Survey. County-level urban vs rural context (urban, metro, and rural) was determined for each patient at diagnosis based on 2013 data from the US Department of Agriculture Economic Research Services. Overall stage was determined from the NCDB Analytic Stage Group variable, which uses the reported pathologic stage, or clinical stage if pathologic stage is unavailable, to classify as stage I, II, III, or IV. Facility types consisted of community cancer, comprehensive community cancer, academic or research, and integrated network cancer programs.

### Outcome

The outcome variable was HPV testing status (tested vs not tested). In the NCDB, HPV testing status is coded within the Collaborative Stage Site-Specific Factor 10 variable for patients diagnosed from 2013 to 2017 and Schema Discriminator 2 variable for those diagnosed from 2018 to 2021 (eFigure 2 in Supplement 1). We designated patients as *tested* if they were coded as being HPV positive or HPV negative or if testing was ordered but the result was not reported. We designated patients as *not tested* if testing was not performed, testing status was unknown, or there was no information on HPV testing status. We combined those who were not tested with those whose testing status was unknown for the earlier subset since the NCDB variable does not distinguish between not tested and unknown status for the 2018 to 2021 subset. The distribution of HPV testing statuses for the 2013 to 2017 subset can be found in the eTable in Supplement 1. We excluded 54 patients whose HPV testing status was coded as *not applicable*.

### Trend Analysis

We assessed trends in HPV testing from 2013 to 2021 by each exposure variable with annual percentage change (APC) through a joinpoint analysis using Joinpoint software, version 5.3 (National Cancer Institute). The methods are described in detail elsewhere.^9^ In brief, this method estimates whether one or more discrete changes (called joinpoints) occurred in an otherwise linear trend. Joinpoints are added to the model if they substantially improve the fit through permutation tests as determined by the program.^10^ We fit the trends across the whole study period and found joinpoints in 2018 or 2019 for all categories of our exposure variables. Thus, we fit the model across 2013 to 2017 and 2018 to 2021. Our response variable was the percentage of patients who were HPV tested.

### Statistical Analysis

Descriptive statistics were generated to compare the proportions of patients who received HPV testing within each category of our exposure variables. The χ^2^ test was used to assess statistical significance for categorical variables, and the Mann-Whitney *U* test was used for age. Poisson regression with full case analysis was performed for both subsets to calculate the adjusted prevalence ratios (PRs) of not being tested for HPV.^11^ We intentionally excluded overall stage in our Poisson regression models because HPV status was clinically used to determine stage for the later subset of patients. Therefore, stage could not be considered a factor associated with HPV testing status. Statistical analysis was performed in 2024 using R, version 3.5.1 (R Foundation for Statistical Computing). All statistical testing was 2-sided with *P* < .05 considered statistically significant.

## Results

### Univariate Analysis

The total population was 135 756 patients with OPSCC (7.3% Black, 3.9% Hispanic, 86.2% White, 2.6% other; 83.4% male; 16.6% female). From 2013 to 2017, a total of 70 911 patients were diagnosed with OPSCC (7.5% Black, 3.7% Hispanic, 86.4% White, and 2.4% other; 82.9% male and 17.1% female). Of these, 50 494 (71.2%) were tested for HPV, whereas 20 417 (28.8%) were not tested for HPV or had unknown testing status. The proportions of patients who were not tested from 2013 to 2017 differed significantly across categories of all variables (Table 1). HPV nontesting rates decreased from 2013 (36.4%) to 2017 (21.9%). Higher proportions of Black patients (36.8%) and uninsured patients (37.4%) were not tested compared with patients of all other races or insurance types. Patients living in underserved areas (zip codes with the lowest educational attainment or lowest median income) had higher proportions of patients not tested (both 35.1%). “Other” subsite had the highest proportion of not tested (39.0%), whereas tonsil subsite had the lowest (24.7%). Community cancer programs had the highest proportion of patients not tested (33.2%), whereas academic or research programs had the lowest (26.6%). The median (IQR) age was 60 (47-73) years for tested patients and 62 (48-76) years for untested patients.

**Table 1.** Descriptive Statistics for 135 756 Patients With Oropharyngeal Cancer Tested and Not Tested for Human Papillomavirus in the National Cancer Database, 2013-2021

Variable	2013-2017 (n = 70 911)					2018-2021 (n = 64 845)			
	Patients, No. (%)^a^				*P* value	Patients, No. (%)^a^			*P* value
	Tested (n = 50 494)	Not tested (n = 20 417)^b^				Tested (n = 60 184)	Not tested (n = 4661)^b^		
**Demographic variables**									
Sex									
Female	8216 (67.8)		3905 (32.2)	<.001		9378 (89.9)		1059 (10.1)	<.001
Male	42 278 (71.9)		16 512 (28.1)			50 806 (93.4)		3602 (6.6)	
Race and ethnicity^c^									
Black	3343 (63.2)		1945 (36.8)	<.001		4140 (90.5)		436 (9.5)	<.001
Hispanic	1743 (67.1)		855 (32.9)			2482 (91.7)		224 (8.3)	
White	44 221 (72.1)		17 073 (27.9)			51 962 (93.1)		3832 (6.9)	
Other^d^	1187 (68.6)		544 (31.4)			1600 (90.5)		169 (9.5)	
Age, median (IQR), y	60 (47-73)		62 (48-76)	<.001		63 (50-76)		64 (49-79)	<.001
**Socioeconomic variables**									
Insurance status									
Uninsured	1675 (62.6)		1001 (37.4)	<.001		1635 (89.7)		187 (10.3)	<.001
Private or managed care	25 354 (76.8)		7663 (23.2)			26315 (94.7)		1472 (5.3)	
Medicaid	4310 (64.8)		2338 (35.2)			5637 (90.9)		562 (9.1)	
Medicare	16 930 (68.0)		7962 (32.0)			23 920 (91.5)		2216 (8.5)	
Other government	1419 (67.2)		694 (32.8)			2031 (92.6)		163 (7.4)	
Zip code median income, $									
<40 227	7019 (64.9)		3789 (35.1)	<.001		8483 (90.6)		884 (9.4)	<.001
40 227-50 353	9165 (68.8)		4154 (31.2)			11 099 (92.4)		911 (7.6)	
50 354-63 332	9924 (71.6)		3934 (28.4)			12 200 (93.3)		876 (6.7)	
≥63 333	16 866 (75.6)		5431 (24.4)			18 981 (93.9)		1239 (6.1)	
Zip code without HS diploma, %									
≥17.6	7627 (64.9)		4132 (35.1)	<.001		8955 (90.6)		933 (9.4)	<.001
10.9-17.5	10 918 (69.2)		4856 (30.8)			13 584 (92.7)		1070 (7.3)	
6.3-10.8	12 499 (72.8)		4677 (27.2)			14 806 (93.2)		1079 (6.8)	
<6.3	12037 (76.5)		3691 (23.5)			13 517 (94.2)		839 (5.8)	
Urban or rural									
Metro	41 002 (71.9)		15 973 (28.1)	<.001		48 541 (92.9)		3688 (7.1)	<.001
Urban	6688 (67.2)		3263 (32.8)			8658 (91.9)		760 (8.1)	
Rural	798 (66.3)		405 (33.7)			1030 (91.9)		91 (8.1)	
**Clinical characteristics**									
Year of diagnosis									
2013	8132 (63.6)		4662 (36.4)	<.001		NA		NA	<.001
2014	9061 (67.3)		4411 (32.7)			NA		NA	
2015	10047 (71.1)		4087 (28.9)			NA		NA	
2016	11 110 (74.3)		3844 (25.7)			NA		NA	
2017	12 144 (78.1)		3413 (21.9)			NA		NA	
2018	NA		NA			14 370 (91.1)		1406 (8.9)	
2019	NA		NA			15 778 (92.8)		1226 (7.2)	
2020	NA		NA			15 188 (93.7)		1026 (6.3)	
2021	NA		NA			14 848 (93.7)		1003 (6.3)	
Subsite									
Base of tongue	19 725 (69.3)		8738 (30.7)	<.001		22 502 (90.9)		2247 (9.1)	<.001
Other	5052 (61.0)		3225 (39.0)			9400 (90.8)		955 (9.2)	
Tonsil	25 717 (75.3)		8454 (24.7)			28 282 (95.1)		1459 (4.9)	
Overall stage^e^									
I	2072 (60.1)		1376 (39.9)	<.001		24 735 (97.8)		558 (2.2)	<.001
II	3193 (68.9)		1442 (31.1)			14 428 (96.7)		488 (3.3)	
III	8023 (72.9)		2975 (27.1)			8381 (93.6)		570 (6.4)	
IV	34 979 (73.1)		12 871 (26.9)			6965 (80.5)		1689 (19.5)	
**Hospital factors**									
Facility type									
Community cancer	2926 (66.8)		1455 (33.2)	<.001		3784 (90.6)		393 (9.4)	<.001
Comprehensive community cancer	15 847 (69.1)		7074 (30.9)			18 859 (92.4)		1558 (7.6)	
Academic or research	22 844 (73.4)		8280 (26.6)			26 746 (93.6)		1842 (6.4)	
Integrated network cancer	8289 (70.8)		3422 (29.2)			10 240 (92.6)		815 (7.4)	

Abbreviations: HS, high school; NA, not applicable.

^a^ Unless otherwise indicated, percentage values are from data in rows.

^b^ Not tested includes those whose human papillomavirus testing status was unknown.

^c^ Race and ethnicity as designated by the National Cancer Database based on patient-reported data.

^d^ Other race and ethnicity based on self-report, including otherwise not specified.

^e^ To calculate overall stage, the seventh edition of the *American Joint Committee On Cancer Staging Manual* was used for patients diagnosed from 2013 to 2017, and the eighth edition was used for patients diagnosed from 2018 to 2021.

From 2018 to 2021, a total of 64 845 patients were diagnosed with OPSCC (7.1% Black, 4.2% Hispanic, 86.0% White, and 2.7% other; 83.9% male and 16.1% female). Of these, 60 184 (92.8%) were tested for HPV, whereas 4661 (7.2%) were not tested for HPV or had unknown testing status. Similar to the earlier subset, HPV nontesting rates decreased substantially from 2018 (8.9%) to 2021 (6.3%), although the magnitude of the decrease was smaller (Table 1). Higher proportions of Black patients (9.5%) and uninsured patients (10.3%) were not tested compared with patients of all other races or insurance types. Patients residing in zip codes with the lowest median income or lowest educational attainment had higher proportions of not tested (both 9.4%). “Other” subsite had the highest proportion of not tested (9.2%), whereas tonsil subsite had the lowest (4.9%). Community cancer programs had the highest proportion of patients not tested (9.4%), whereas academic or research programs had the lowest (6.4%). The median (IQR) age was 63 (50-76) years for tested patients and 64 (49-79) years for untested patients.

### Trend Analysis

HPV testing rates increased from 2013 to 2017 across categories of all exposure variables, whereas from 2018 to 2021 many groups’ testing rates plateaued (Table 2). HPV testing rates increased annually by 6.8% (95% CI, 4.5%-9.8%) from 2013 to 2017 and by 1.2% (95% CI, 0.5%-2.1%) from 2018 to 2021 among Black patients compared with 4.9% (95% CI, 4.5%-5.4%) from 2013 to 2017 and 0.8% (95% CI, −0.2% to 2.0%) from 2018 to 2021 among White patients (Figure, A). From 2018 to 2021, the HPV testing rate APCs plateaued for patients living in zip codes with the lowest educational attainment (1.0%; 95% CI, −0.6% to 2.8%) (Figure, B) and the lowest median income (1.2%; 95% CI, −0.3% to 2.8%) (Figure, C). Among patients with Medicaid insurance, the HPV testing rate increased annually by 7.9% (95% CI, 7.4%-8.5%) from 2013 to 2017 and by 1.6% (95% CI, 0.2%-3.3%) from 2018 to 2021 (Figure, D). The HPV testing rate increased annually by 4.3% (95% CI, 3.5%-5.3%) from 2013 to 2017 and by 0.7% (95% CI, 0.4%-1.1%) from 2018 to 2021 for academic or research programs (Figure, E). Lastly, the HPV testing rate APCs plateaued from 2018 to 2021 for all urban and rural categories (Figure, F).

**Table 2.** Joinpoint Annual Percentage Changes for Human Papillomavirus Testing Rates

Variable	Annual percentage change (95% CI)	
	2013-2017	2018-2021
Facility type		
Community	7.4 (3.7 to 12.3)	1.3 (−1.5 to 4.6)
Comprehensive community	6.2 (4.0 to 9.0)	1.2 (−0.5 to 3.1)
Academic or research	4.3 (3.5 to 5.3)	0.7 (0.4 to 1.1)
Integrated network	4.8 (4.1 to 5.6)	0.7 (0.07 to 1.4)
Zip code without HS diploma, %		
≥17.6	8.4 (7.3 to 9.9)	1.0 (−0.6 to 2.8)
10.9-17.5	6.1 (5.8 to 6.5)	1.1 (0.6 to 1.8)
6.3-10.8	5.2 (4.9 to 5.5)	0.8 (−0.8 to 2.6)
<6.3	3.7 (1.6 to 6.3)	0.7 (0.66 to 0.73)
Zip code median annual income, $		
<40 227	7.4 (6.0 to 9.2)	1.2 (−0.3 to 2.8)
40 227-50 353	6.1 (5.5 to 6.8)	0.8 (0.5 to 1.1)
50 354-63 332	6.1 (5.2 to 7.2)	0.8 (−0.2 to 1.8)
≥ 63 333	4.3 (3.0 to 5.9)	1.0 (−0.3 to 2.4)
Insurance status		
Not insured	7.2 (4.3 to 10.4)	0.8 (−0.4 to 2.0)
Private or managed care	3.5 (1.9 to 5.3)	0.7 (−0.3 to 1.9)
Medicaid	7.9 (7.4 to 8.5)	1.6 (0.2 to 3.3)
Medicare	5.6 (4.6 to 6.8)	0.9 (−0.1 to 2.0)
Other government	9.5 (7.0 to 13.3)	1.9 (0.4 to 3.6)
Race and ethnicity^a^		
Black	6.8 (4.5 to 9.8)	1.2 (0.5 to 2.1)
Hispanic	8.2 (7.4 to 9.4)	1.1 (−2.2 to 4.8)
White	4.9 (4.5 to 5.4)	0.8 (−0.2 to 2.0)
Other^b^	5.1 (1.6 to 9.9)	1.4 (−0.2 to 3.2)
Urban or rural		
Metro	4.8 (4.5 to 5.2)	1.0 (−0.1 to 2.2)
Urban	7.0 (6.5 to 7.7)	0.7 (−0.3 to 1.8)
Rural	6.7 (3.0 to 11.1)	0.2 (−2.4 to 3.1)

Abbreviation: HS, high school.

^a^ Race and ethnicity as designated by the National Cancer Database based on patient-reported data.

^b^ Other race and ethnicity based on self-report, including otherwise not specified.

**Figure.**
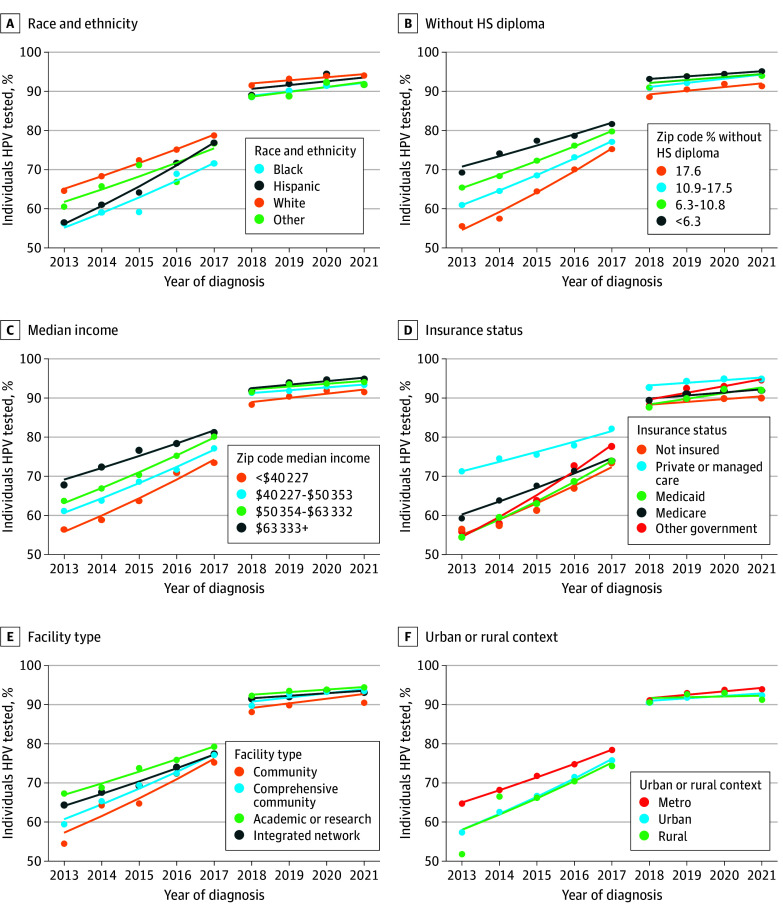
Trends in Human Papillomavirus (HPV) Testing From 2013 to 2021 A, Race and ethnicity as designated by the National Cancer Database based on patient-reported data. Other race and ethnicity based on self-report, including otherwise not specified. HS indicates high school.

### Multivariable Analysis

We calculated adjusted PRs of not being tested for HPV for the 2013 to 2017 and 2018 to 2021 subsets (Table 3). Among patients diagnosed from 2013 to 2017, Black patients (PR, 1.19; 95% CI, 1.12-1.25), Hispanic patients (PR, 1.11; 95% CI, 1.02-1.20), and patients of other races (PR, 1.16; 95% CI, 1.05-1.28) were more likely not to be tested for HPV compared with White patients, holding all other factors constant. Patients whose cancer cases were recorded at community (PR, 1.25; 95% CI, 1.18-1.33), comprehensive community (PR, 1.20; 95% CI, 1.15-1.24), or integrated network (PR, 1.17; 95% CI, 1.12-1.23) cancer programs were more likely not to be tested for HPV compared with academic or research programs. Patients who were uninsured (PR, 1.45; 95% CI, 1.35-1.56) or had Medicaid (PR, 1.36; 95% CI, 1.29-1.44), Medicare (PR, 1.30; 95% CI, 1.25-1.34), or other government (PR, 1.35; 95% CI, 1.24-1.47) insurance were more likely not to be tested for HPV compared with patients with private insurance or managed care. Patients with base of tongue (PR, 1.23; 95% CI, 1.19-1.27) or other (PR, 1.46; 95% CI, 1.40-1.53) subsite were more likely not to be tested for HPV compared with tonsil subsite.

**Table 3.** Poisson Regression Analysis for Adjusted PRs of Not Being Tested for Human Papillomavirus^a^

Variable	2013-2017			2018-2021		
	Adjusted PR (95% CI)	*P* value		Adjusted PR (95% CI)	*P* value	
		Compared with reference category	Global		Compared with reference category	Global
Sex						
Male	1 [Reference]	NA	<.001	1 [Reference]	NA	<.001
Female	1.10 (1.06-1.15)	<.001		1.49 (1.38-1.61)	<.001	
Race and ethnicity^b^						
White	1 [Reference]	NA	<.001	1 [Reference]	NA	<.001
Black	1.19 (1.12-1.25)	<.001		1.18 (1.05-1.33)	.005	
Hispanic	1.11 (1.02-1.20)	.01		1.15 (0.99-1.34)	.08	
Other^c^	1.16 (1.05-1.28)	.003		1.39 (1.17-1.66)	<.001	
Insurance status						
Private	1 [Reference]	NA	<.001	1 [Reference]	NA	<.001
Uninsured	1.45 (1.35-1.56)	<.001		1.69 (1.42-2.00)	<.001	
Medicaid	1.36 (1.29-1.44)	<.001		1.45 (1.29-1.62)	<.001	
Medicare	1.30 (1.25-1.34)	<.001		1.44 (1.34-1.55)	<.001	
Other government	1.35 (1.24-1.47)	<.001		1.38 (1.16-1.66)	<.001	
Year of diagnosis						
2013	1.66 (1.58-1.75)	<.001	<.001	NA	NA	<.001
2014	1.49 (1.42-1.57)	<.001		NA	NA	
2015	1.36 (1.29-1.43)	<.001		NA	NA	
2016	1.18 (1.12-1.24)	<.001		NA	NA	
2017	1 [Reference]	NA		NA	NA	
2018	NA	NA		1.46 (1.33-1.60)	<.001	
2019	NA	NA		1.17 (1.06-1.28)	.001	
2020	NA	NA		1.04 (0.94-1.14)	.47	
2021	NA	NA		1 [Reference]	NA	
Subsite						
Tonsil	1 [Reference]	NA	<.001	1 [Reference]	NA	<.001
Base of tongue	1.23 (1.19-1.27)	<.001		1.83 (1.70-1.97)	<.001	
Other	1.46 (1.40-1.53)	<.001		1.78 (1.63-1.95)	<.001	
Zip code without HS diploma, %						
<6.3	1 [Reference]	NA	<.001	1 [Reference]	NA	<.001
≥17.6	1.24 (1.16-1.31)	<.001		1.33 (1.17-1.52)	<.001	
10.9-17.5	1.16 (1.10-1.23)	<.001		1.11 (0.99-1.24)	.07	
6.3-10.8	1.10 (1.05-1.15)	<.001		1.12 (1.01-1.24)	.02	
Zip code median income, $						
≥63 333	1 [Reference]	NA	.004	1 [Reference]	NA	.007
<40 227	1.09 (1.03-1.16)	.004		1.17 (1.04-1.33)	.01	
40 227-50 353	1.07 (1.02-1.13)	.01		1.06 (0.95-1.18)	.26	
50 354-63 332	1.04 (1.00-1.09)	.07		0.97 (0.88-1.08)	.61	
Facility type						
Academic or research	1 [Reference]	NA	<.001	1 [Reference]	NA	<.001
Community cancer	1.25 (1.18-1.33)	<.001		1.36 (1.20-1.54)	<.001	
Comprehensive community cancer	1.20 (1.15-1.24)	<.001		1.20 (1.11-1.29)	<.001	
Integrated network cancer	1.17 (1.12-1.23)	<.001		1.17 (1.06-1.28)	.001	
Urban or rural						
Urban	1 [Reference]	NA	<.001	1 [Reference]	NA	.86
Metro	0.93 (0.89-0.97)	.002		0.98 (0.89-1.07)	.64	
Rural	1.02 (0.91-1.14)	.76		0.95 (0.75-1.22)	.70	

Abbreviations: HS, high school; NA, not applicable; PR, prevalence ratio.

^a^ Only patients with nonmissing values for all variables were included in the Poisson regression models. Not tested includes those whose human papillomavirus testing status was unknown.

^b^ Race and ethnicity as designated by the National Cancer Database based on patient-reported data.

^c^ Other race and ethnicity based on self-report, including otherwise not specified.

Among patients diagnosed from 2018 to 2021, Black patients (PR, 1.18; 95% CI, 1.05-1.33) and patients of other races (PR, 1.39; 95% CI, 1.17-1.66) were more likely not to be tested for HPV compared with White patients, holding all other factors constant. Patients whose cancer cases were recorded at community (PR, 1.36; 95% CI, 1.20-1.54), comprehensive community (PR, 1.20; 95% CI, 1.11-1.29), or integrated network (PR, 1.17; 95% CI, 1.06-1.28) cancer programs were more likely not to be tested for HPV compared with academic or research programs. Patients who were uninsured (PR, 1.69; 95% CI, 1.42-2.00) or had Medicaid (PR, 1.45; 95% CI, 1.29-1.62), Medicare (PR, 1.44; 95% CI, 1.34-1.55), or other government (PR, 1.38; 95% CI, 1.16-1.66) insurance were more likely not to be tested for HPV compared with patients with private insurance or managed care. Patients with base of tongue (PR, 1.83; 95% CI, 1.70-1.97) or other (PR, 1.78; 95% CI, 1.63-1.95) subsite were more likely not to be tested for HPV compared with tonsil subsite.

## Discussion

The current study is the first, to our knowledge, to investigate HPV testing rates and factors associated with HPV testing in OPSCC after 2017. Our results highlight substantial socioeconomic and clinical disparities in HPV testing as late as 2021. Specifically, race other than White, lower income and educational attainment by zip code, nonprivate insurance, subsite other than tonsil, and nonacademic cancer programs were independently associated with decreased likelihood of having HPV testing between 2018 and 2021. These findings align with prior literature documenting health disparities on a larger scale and more specifically in head and neck cancer.^12^

Similar to studies^7,8^ before 2018, our study found that patients of Black, Hispanic, and other race or ethnicity were less likely to be tested for HPV than White patients. Although the adjusted PR for not being HPV tested for Black patients was similar in the earlier and later years, the testing disparity for patients of other race or ethnicity widened over time. Racial disparities in the delivery of health care are well documented in the US and are driven by a combination of individual and systemic factors.^13^ Historical evidence that Black patients are less likely to have HPV-positive OPSCC may make clinicians less inclined to pursue HPV testing for Black patients compared with their White counterparts.^14^ Furthermore, implicit bias and stereotyping of patients of races other than White may subconsciously affect clinicians’ judgment and decision-making.^15,16^ These biases, together with structural racism, may be factors in systematic inequities in how preventive and diagnostic tools, including HPV testing, are used across racial groups.^17,18^ Bias training and interventions addressing structural racism may reduce racial disparities in HPV testing for OPSCC.^19^

Social determinants of health likely play a role in HPV testing disparities. For example, health literacy may explain our finding with educational attainment at the zip code level. Research has shown that low patient health literacy is prevalent among racial minority groups and is associated with neighborhood deprivation.^20^ Limited understanding of HPV and its association with OPSSC may hinder patients from engaging in conversations about HPV testing with their clinicians. Therefore, improving patient education, facilitating communication, and increasing health literacy can help address HPV testing disparities. Additionally, insurance status plays a critical role in exacerbating health disparities in the US because health insurance may be inaccessible to those who are unemployed or cannot afford insurance.^21,22^ Because insurance status is associated with treatment modality and patient outcomes in head and neck cancer,^21^ it likely also plays a role in HPV testing for OPSCC. Therefore, policy changes and health insurance reform could reduce HPV testing disparities in OPSCC.

Our multivariable analysis revealed that facility type was independently associated with HPV testing for both the 2013 to 2017 and 2018 to 2021 subsets. At a systemic level, prior research^23^ has shown that community cancer centers are potential factors in health disparities because patients are less likely to receive guideline-recommended care and experience worse outcomes compared with those treated at academic medical centers or National Cancer Institute–designated cancer centers. A 2021 study^23^ found that using an academic and community collaboration model at community cancer centers effectively increased adherence to quality measures. Because community cancer centers are estimated to treat approximately 80% to 85% of patients with cancer in the US,^23^ targeted interventions to encourage guideline adherence and increase resources can help reduce HPV testing disparities.

Of all subsites, patients with tonsillar cancer were the most likely to be tested. Because tonsillar cancer is the most common type of HPV-positive OPSCC and its association with HPV is well recognized,^24^ clinicians may be less likely to pursue HPV testing for other subsites, including base of tongue. Therefore, raising awareness on the role of HPV in nontonsillar subsites may help increase HPV testing rates for these potentially overlooked sites.

Notably, no subset of patients diagnosed between 2018 and 2021 experienced testing rates near 100%. Substandard HPV testing is especially concerning given that the incidence rates of OPSCC are increasing in the US and that the current staging requires HPV status (*AJCC 8*).^6^ Beyond informing treatment selection, HPV status is necessary for nearly all prospective research. Therefore, patients who are not tested for HPV may be disproportionately excluded from participating in clinical trials. Because members of racial and ethnic minoritized groups have historically been underrepresented in clinical trials in the US, inequitable HPV testing in OPSCC is possibly a factor in their exclusion.^25^

### Limitations

This study has several limitations. Selection bias is possible because patients included in the NCDB may be systematically different from those excluded, but the NCDB covers a large proportion of the population by collecting data on approximately 70% of incident cancer cases in the US.^8^ Outcome misclassification bias is possible due to inherent limitations with the clinical methods used for HPV testing. For example, biopsy samples procured using fine needle aspiration may be inadequate to accurately determine HPV status and be classified as not tested or unknown.^26^ However, we do not expect this to be differential with respect to our exposures, thus biasing our results to the null.^27^ Our exposure variables of median income and educational attainment were designated by the NCDB at the zip code level, limiting our ability to investigate these metrics of socioeconomic status at the individual level. Limitations with the HPV testing status variable itself prevented us from determining which patients diagnosed with OPSCC from 2018 to 2021 did not receive testing vs had unknown testing status. However, by grouping patients diagnosed from 2013 to 2017 whose HPV testing status was unknown with those who were not tested, we were able to consistently compare testing for the earlier and later subsets.

## Conclusions

To our knowledge, the current study represents the first analysis of HPV testing rates and trends since the implementation of the *AJCC 8* in 2018. We found that HPV testing rates remained substandard in 2021, almost a decade after the 2012 NCCN recommendation that every case of OPSCC be tested for HPV. Although HPV testing rates improved over time, sociodemographic and clinical variables were still associated with the likelihood of HPV testing between 2018 and 2021, with Black patients and those with cancer cases recorded at community cancer programs less likely to receive HPV testing compared with White patients and those at academic or research programs. Further research is needed to corroborate these findings, identify barriers to HPV testing, and develop interventions to increase the uptake of HPV testing in OPSCC.

## Notes

Supplement 1. **eFigure 1.** CONSORT Diagram of Study Sample **eFigure 2.** Flowchart Describing the HPV Testing Status Variables in the National Cancer Database (NCDB) For Our 2013-2017 Versus 2018-2021 Subsets **eTable.** Distribution of HPV Testing Statuses, as Indicated By the NCDB CS-Site Specific Factor 10 Variable, for the 2013-2017 Subset (n = 70911)

Supplement 2.
Data Sharing Statement
