# Supplementary material for: Trends in Human Papillomavirus Testing Among Patients With Oropharyngeal Cancer
Source: JAMA Netw Open. 2025 Jul 29;8(7):e2523917. doi: 10.1001/jamanetworkopen.2025.23917 (PMC12308448; doi:10.1001/jamanetworkopen.2025.23917)
Supplement: Supplement 1. — eFigure 1. CONSORT Diagram of Study Sample eFigure 2. Flowchart Describing the HPV Testing Status Variables in the National Cancer Database (NCDB) For Our 2013-2017 Versus 2018-2021 Subsets eTable. Distribution of HPV Testing Statuses, as Indicated By the NCDB CS-Site Specific Factor 10 Variable, for the 2013-2017 Subset (n = 70911) [file jamanetwopen-e2523917-s001.pdf]

Supplemental Online Content

Carlson KM, Abdul-Rahman N-H, Deek RA, et al. Trends in human papillomavirus testing among patients with oropharyngeal cancer. *JAMA Netw Open*. 2025;8(7):e2523917. doi:10.1001/jamanetworkopen.2025.23917

eFigure 1. CONSORT Diagram of Study Sample

eFigure 2. Flowchart Describing the HPV Testing Status Variables in the National Cancer Database (NCDB) for Our 2013-2017 Versus 2018-2021 Subsets

eTable. Distribution of HPV Testing Statuses, as Indicated by the NCDB CS-Site Specific Factor 10 Variable, for the 2013-2017 Subset (n=70911)

This supplemental material has been provided by the authors to give readers additional information about their work.

© 2025 Carlson KM et al. *JAMA Network Open.*

eFigure 1. CONSORT diagram of study sample

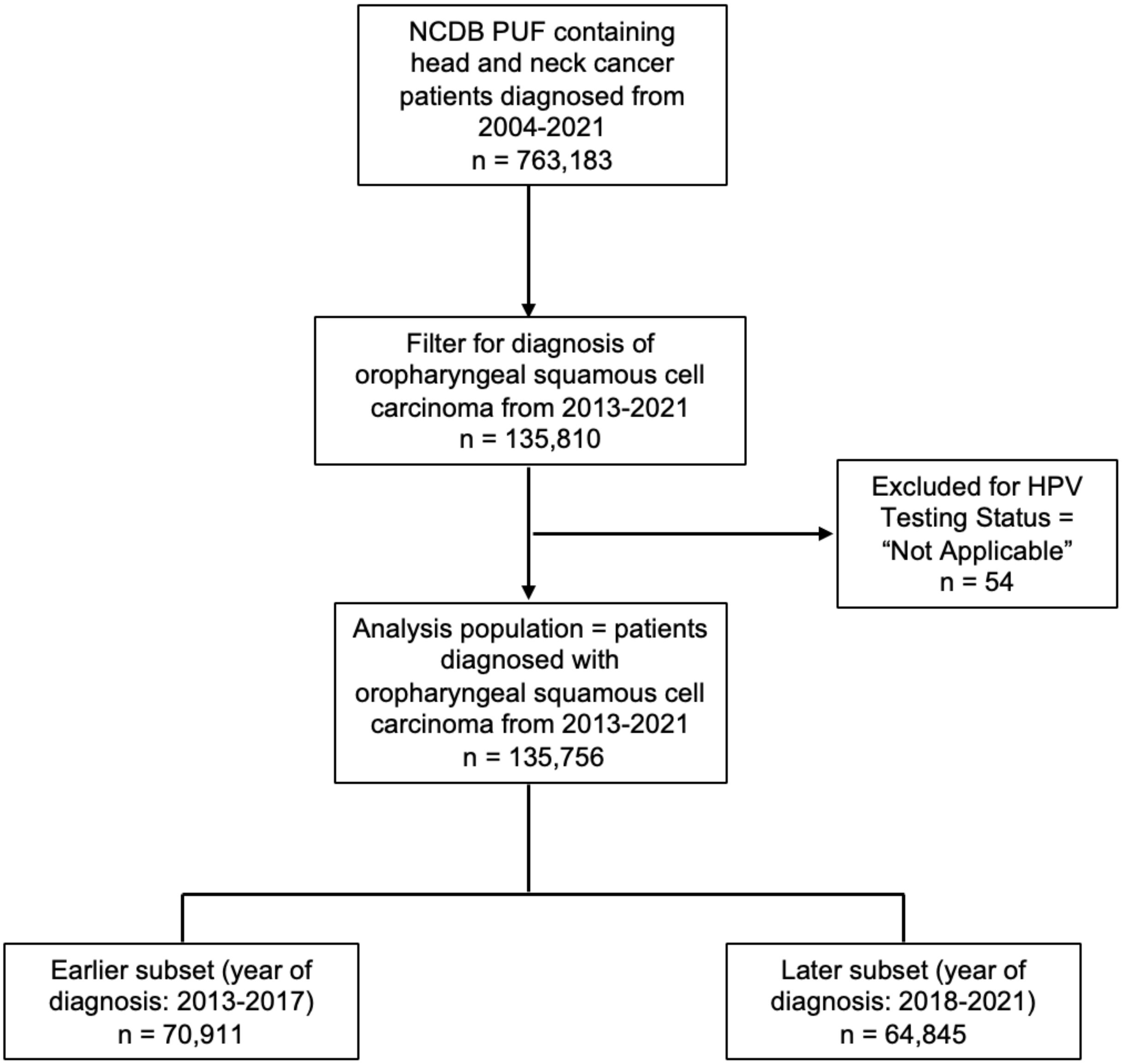

© 2025 Carlson KM et al. *JAMA Network Open.*

eFigure 2. Flowchart describing the HPV testing status variables in the National Cancer Database (NCDB) for our 2013-2017 versus 2018-2021 subsets

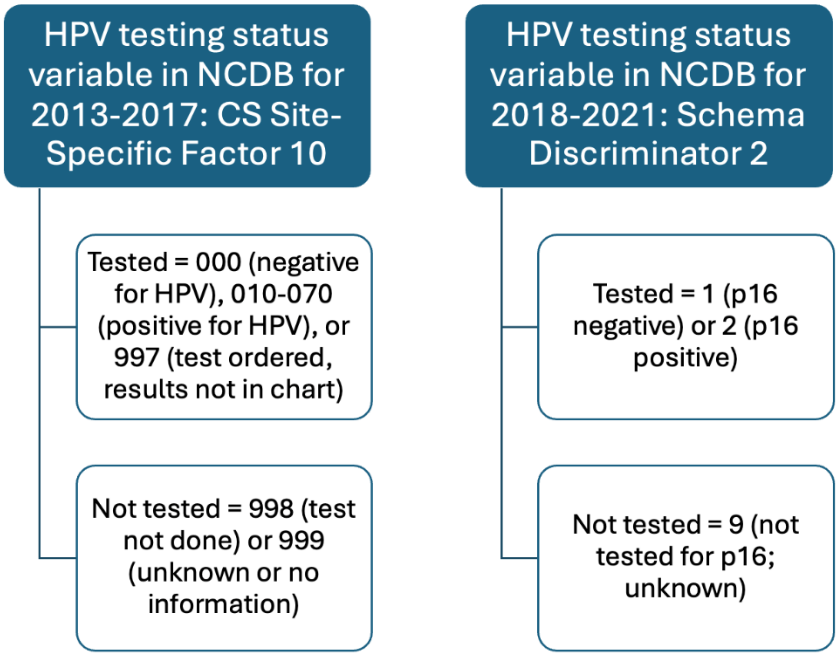

© 2025 Carlson KM et al. *JAMA Network Open.*

eTable. Distribution of HPV testing statuses, as indicated by the NCDB CS-Site Specific Factor 10 variable, for the 2013-2017 subset (n=70911)

**Tested**		**Not Tested**		**Unknown testing status**	
Codes	Number (%)	Code	Number (%)	Code	Number (%)
000, 010, 020, 030, 040, 050, 060, 070, 997	50494 (71.2)	998	5891 (8.3)	999	14526 (20.5)

© 2025 Carlson KM et al. *JAMA Network Open.*
