# Supplementary material for: Trends in Human Papillomavirus Testing Among Patients With Oropharyngeal Cancer
Source: JAMA Netw Open. 2025 Jul 29;8(7):e2523917. doi: 10.1001/jamanetworkopen.2025.23917 (PMC12308448; doi:10.1001/jamanetworkopen.2025.23917)
Supplement: Supplement 2. — Data Sharing Statement [file jamanetwopen-e2523917-s002.pdf]

Data Sharing Statement

Carlson. Trends in Human Papillomavirus Testing Among Patients With Oropharyngeal Cancer. *JAMA Netw Open*. Published July 29, 2025.
doi:10.1001/jamanetworkopen.2025.23917

Data
Data available: No

Additional Information
Explanation for why data not available: Participant user files from the National Cancer Database are available to investigators associated with Commission on Cancer-accredited cancer programs. Requests for access to the statistical/analytic code used in this study can be made to the corresponding author, Dr. Angela L. Mazul (angela.mazul@pitt.edu).
